# Biomechanical Properties of Heel Pad, Metatarsal Head Soft-Tissue and Foot Ulcers in Patients with Systemic Sclerosis: A Case Control Study

**DOI:** 10.31138/mjr.33.1.35

**Published:** 2022-03-31

**Authors:** Mandana Pourian, Iman Mohseni, Elham Andalib, Hadi Poormoghim

**Affiliations:** 1 Radiology Department, Firoozgar Hospital, Iran University of Medical Sciences, Tehran, Iran,; 2 Rheumatology Department, Firoozgar Hospital, Iran University of Medical Sciences, Tehran, Iran

**Keywords:** systemic sclerosis, ultrasonography, compressibility index, scleroderma foot, heel pad

## Abstract

**Background:**

Systemic sclerosis is a chronic disease of connective tissue accompanied by fibrosis of the skin and inner organs and an increased risk of foot ulcers. Biomechanical indices such as soft-tissue thickness and compressibility may correlate with the risk of this phenomenon.

**Objective:**

The aim of this study was to assess heel pad and first metatarsal head (MTH) soft-tissue thickness and compressibility index (CI) in scleroderma patients compared to matched healthy individuals. Not all patients had foot ulcers.

**Methods:**

Heel pad thickness in standing (loaded) and lying (unloaded) positions were measured in 40 scleroderma patients by means of a lateral foot radiograph. CI was measured as the ratio of loaded to unloaded thickness. The Soft-tissue thickness of the first MTH was measured by ultrasound. Results were compared with 40 healthy controls of matched age and body mass index. All patients’ diagnoses were made based on the American College of Rheumatology classification criteria.

**Results:**

Forty scleroderma patients (36 females, 4 males) with the following demographics were studied; mean age (SD) 45(12), mean body mass index 25.5 (4), and mean disease duration=10(9.6) years; only 8 (20%) had digital ulcers. Patients’ heel pad thickness and CI in the dominant side and MTH soft-tissue thickness on both sides were significantly different compared to the control group. Comparison of results in patients with and without foot ulcers also showed a significant difference in soft-tissue thickness. Thickness was negatively associated with disease duration, but the CI did not change over time.

**Conclusion:**

Soft-tissue thickness of the foot decreases in scleroderma patients and is associated with foot ulcers and digital ulcers in the hands.

## INTRODUCTION

Systemic sclerosis (SSc) is a connective tissue disorder characterised by three principal features: vasculopathy, immune, and fibroblast dysfunction with excessive matrix deposition leading to fibrosis of the skin and internal organs.^1,2^ In systemic sclerosis, hand lesions are well recognised and represent a vasculopathy feature of the disease.^3^ A limited number of studies have addressed the podiatric complications of SSc. In patients with SSc, foot vasculopathy presents as Raynaud’s phenomenon, telangiectasia, digital scar and/or ulcer, gangrene, and amputation.^4,5^ Non-vascular foot lesions (mechanical/pressure-related lesions) present as callus and corn lesions.^5^ Foot ulcerations have been reported in 26%–35%, callus formations in 40–80%, and calcinosis in 18% of SSc patients.^4,5^

Many factors can cause foot pain and lesions. The known causes include changes in skin thickness on the plantar surface, fat pad atrophy, and subclinical neuropathic changes in the foot due to vasculopathy of small neuro-vasorum vessels.^5^ To our knowledge, to date, no study has addressed the biomechanical changes taking place in SSc patients’ feet.

The primary goal of the current study was to evaluate changes in soft-tissue thickness and stiffness (defined as Compressibility Index) of both heel pads and first metatarsal head (MTH) in SSc patients. In order to evaluate foot ulcers in the course of the disease, we used inexpensive and widely used imaging methods such as ultrasonography (USG) and radiography.

## PATIENTS AND METHODS

### Study design

Our study was a cross-sectional survey of the patients attending the Rheumatology Clinic of Firoozgar Hospital from October 2016 to March 2017.

### Study population

Forty patients with systemic sclerosis, fulfilling the diagnostic criteria of the American College of Rheumatology (ACR), and 40 healthy age- and BMI-matched individuals were enrolled. Patients who had other underlying diseases that could affect the biomechanical properties of the foot, eg, history of diabetes mellitus, heart failure or coronary artery disease, foot deformity, smoking, peripheral vascular disease were excluded. In this study, non-digital ulcers in the sole are referred to as foot ulcers. Details of the foot ulcers have been reported in another article.^5^ Informed consent was taken from all participants prior to the study. Clinical data and past disease history were drawn from the patients’ medical records.

### Image acquisition

Lateral foot radiographs of both feet (with a film to focus distance of 40 inches using 45 KV and 5 MAS [125 mA, 0.04 sec]) were utilized for evaluating both the unloaded (lying) and loaded (standing) positions of heel pad soft-tissue thickness in both patients and healthy subjects. Loaded radiographs were obtained after 8 seconds of weight bearing on the target foot in standing position.^6^ The soft-tissue density under the surface of the calcaneus was measured to determine the heel pad thickness. The measurement was made from the lowest part of the plantar tuberosity of the calcaneus vertically to the skin edge.

**Figure 1.**
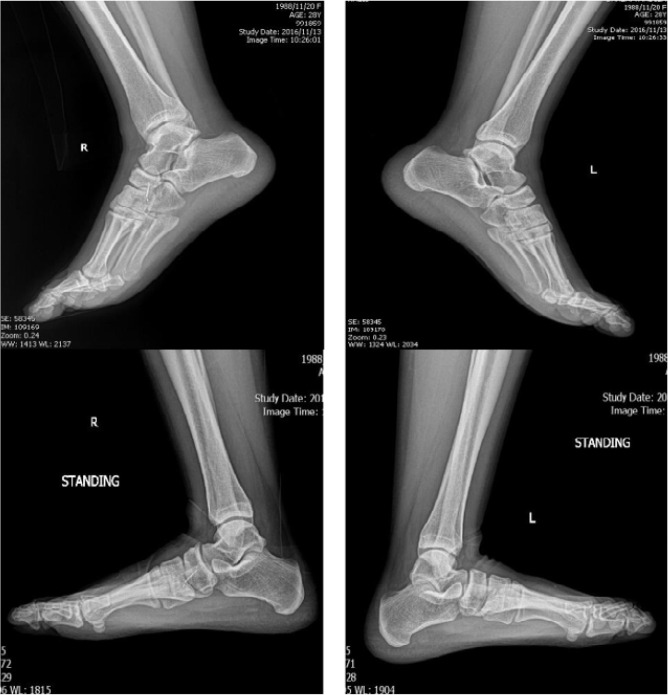
Lateral foot X-ray of both feet in lying (unloaded) and standing (loaded) position.

The compressibility index (CI) was defined as the ratio of the difference of heel pad thickness in loaded to unloaded positions to unloaded thickness. As the index approaches one, the elasticity approaches zero. All measurements were conducted by one radiologist (MP). The radiographic images of a sample patient are shown in **Figure 1**.

Ultrasonographic study of the first MTH was conducted to measure the soft-tissue thickness.^7^ Given the limitations of radiography and overlapping of soft tissue on MTHs in the lateral foot X-ray, we used ultrasound for this part of the study (each subject was placed in the sitting position with ankle in the neutral and knee in the extended position).

**Figure 2.**
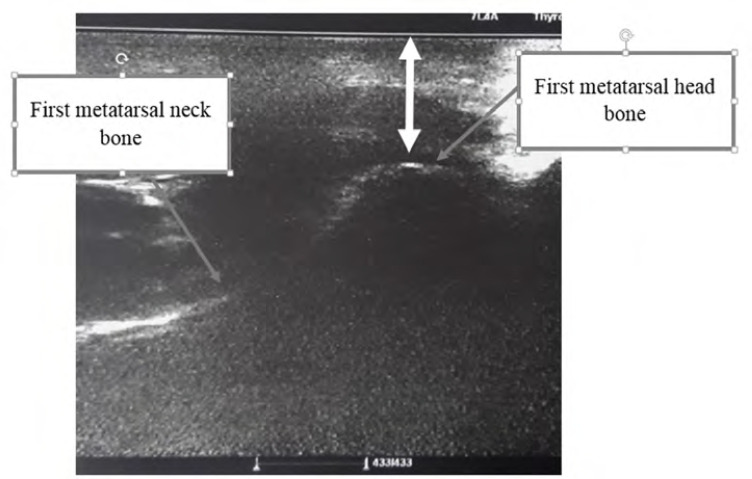
Measurement of first metatarsal head soft tissue thickness with ultrasound.

All measurements were made using the Mindray DC-7 ultrasound machine with 7MHz transducer. Adequate amount of stand-off jelly was applied in order to prevent transducer pressure impairment of the evaluation. Soft-tissue thickness, which was the shortest distance between the first MTH and the skin surface was measured. All measurements were taken by the same researcher. (**Figure 2**)

For comparing heel pad thickness (HPT) of both feet (dominant and non-dominant) and heel pad CI of both feet, the lateral foot radiograph was used on heel. The soft tissue thickness of the first MTH of both feet was measured by USG. These variables, as well as demographic data, were compared in the control and study groups. We conducted correlation analyses to assess the relationships between HPT and compressibility, and demographic and baseline variables.

### Statistical Analysis

Statistical analysis was performed using SPSS 18. We used Chi-square to compare nominal and categorical variables. An independent sample T-test was used for quantitative measurements.

## RESULTS

Mean age (standard deviation=SD) was similar in the study and control groups: 45 (12.3). The mean disease duration was 10 (9.6) years and body mass index (BMI) was 25.5 (3.8) in the study group. In the control group mean age (SD) was 42 (11.5) years and BMI was 26.1 (3.7).

Subjective Raynaud’s phenomenon was observed in 80% of patients and 20% had ulcers. The right foot was the dominant one in most patients. Demographic data and disease characteristics are summarised in **Table 1**.

**Table 1.** Demographic and clinical features of forty SSc patients.

**Character**	^*^NO (%)
Age means (SD), disease	45 (12.3)
Subtype diffuse: limited	25:15
Female: male, disease	36:4
BMI mean (SD), disease	25.5(3.8)
Vascular	
Objective Raynaud	23(57.5%)
Subjective Raynaud	32(80%)
Telangiectasia on hands/face/lips	32(80%)
Dig pitting scar	13(32.5%)
Digital ulcer	8(20%)
Calcinosis	8(20%)
Sclerodactyly	29(72.5)
Friction Rub	12(30%)
Lung	
Lung fibrosis >20%	26(65%)
PAP > 40 on echocardiography	30.5(10.4)
FVC< 70%	11(27.5%)
Dlco <60 %	23(57.5%)
Musculoskeletal	
Arthritis	7(17.5%)
Myositis	7(17.5%)
Foot ulcer	8(20%
Lab	
ANA+	38(95% )
SCL70+	26(65%)
ACA+	6(15%)

* NO(%): Number, percentage

### Heel pad thickness and compressibility index measurement between normal subject and SSc patients

We demonstrated that HPT (17.8 vs 19.5mm) and first MTH soft tissue thickness (10.5 vs 12.5 mm) was significantly lower in patients’ dominant feet when compared to healthy subjects. The compressibility index was significantly higher in SSc patients than in healthy individuals (P value<0.05). (**Table 2**)

**Table 2.** Comparison of thickness and compressibility heel and 1^st^ MTP between normal subjects and SSc patients.

	**SSc patients=40**	**Control=40**	**P-value**
Right heel unloaded Radiograph (mm)	17.80(3.03)	19.5(1.91)	0.03
Right heel compressibility index, CI	0.70(0.076)	0.65(0.087)	0.005
Left heel unloaded Radiograph (mm)	17.96(3.45)	19.0(1.95)	0.1
Left heel compressibility index, CI	0.68(0.08)	0.65(0.08)	0.28
Right metatarsal head US	10.74(2.04)	12.5(1.65)	<0.0001
Left metatarsal head US	10 (1.95)	12.11(1.50)	<0.0001

HPT in scleroderma patients was expected to be 6% less than in healthy subjects (17.8 vs 19.5mm). The overall difference in CI was 9% (0.65 in healthy subjects vs 0.70 in scleroderma patients).

### Heel pad thickness and compressibility index measurement between SSc patients with and without (foot) digital ulcers

Among SSc patients with or without foot ulcers, a significant difference was observed in the HPT of both feet: 15.5 vs 18.4 in the right foot, and 15.0 vs 18.7 in the left foot. However, MTH thickness and compressibility index did not differ between the two groups. (**Table 3**)

**Table 3.** Comparison of thickness and compressibility heel and 1^st^ MTP between SSc patients with and without (foot) digital ulcers (mean).

	**SSc patients with ulcer=8**	**SSc patients without ulcer=32**	**P-value**
Right heel unloaded radiograph	15.5(3.10)	18.7(2.33)	0.01
Right heel compressibility index, CI	0.71(0.075)	0.7(0.072)	0.59
Left heel unloaded radiograph	15(2.82)	18.7(3.23)	0.007
Left heel compressibility index, CI	0.71(0.07)	0.67 (0.07)	0.18
Right metatarsal head US	10.80(2.42)	10.7(1.97)	0.86
Left metatarsal head US	9.34(2.76)	10.20(1.71)	0.27

### Heel pad thickness and compressibility index measurement between SSc patients without foot ulcer and healthy subjects

When we compared patients without ulcers with healthy subjects the CI increased (0.70 vs 0.65, p=0.02), but the HPT decreased without any significant difference (18.4 vs 19.5, p = 0.28).

Among the demographic and baseline factors evaluated in this study, BMI demonstrated positive correlation and disease duration showed negative correlation with HPT. None of the demographic and baseline variables were significantly correlated with CI. Gender and age had no significant correlation or association with any of other variables (**Supplementary Table**).

## DISCUSSION

Foot ulcers are one of the most uncomfortable complications of scleroderma, greatly affecting patients’ functional abilities. This study aimed to focus on the mechanical aspects of this problem.

Using ultrasound imaging, we observed significant changes in the compressibility and elasticity indices and heel pad thickness of scleroderma patients’ feet. Besides our main findings, we observed two additional points. First, in line with the study of Sunderkötter et al., we found a correlation between Raynaud’s phenomenon and ulcer manifestation (P-value 0.006).^8^ However, Raynaud’s phenomenon was not significantly correlated with the quantitative parameters (thickness and compressibility index). This finding may be justified by the vasculopathy nature of both digital ulcers and Raynaud’s phenomenon.

Secondly, in a comparison between patients without ulcers and healthy subjects, we observed that CI had increased, but thickness had not significantly differed. This could mean that changes in stiffness occur prior to changes in thickness, or that, there is no linear correlation between skin thickness and stiffness.

These results could be used in patients’ clinical follow-up, and compressibility changes could be made more valuable to clinicians in their first visits. Nevertheless, as the disease progresses in severity over time, following patients with thickness measurements (mainly that of the heel pad) could prove more prognostic of heel and foot ulcers. Unfortunately, we could not find a cut-off point for thickness from which we can predict a significantly increased risk of digital ulcers. It may thus, be more valuable to compare measurements between visits.

Though we thoroughly searched the literature, we found no similar study that had been conducted on foot biomechanics in scleroderma patients. Foot biomechanics was the main purpose of studies related to other diseases with podiatric soft-tissue involvement, the most important of which is diabetes mellitus (DM). Most studies had used ultrasound as the method of measurement. Chao et al. found a 6% increase in soft-tissue thickness in pure diabetic patients, whereas, 9% and 15% decreases were observed in neuropathic and ulcerated patients, respectively.^9^ Additionally, they found increased stiffness in people with diabetes, particularly in persons affected with neuropathy or ulceration. Although we know that DM and scleroderma share some pathophysiological processes, including vasculopathy and the vascular involvement of digital ulcers, they are not completely the same, as DM does not include fibrinogenic mechanisms. Ultrasound (US) is a cost-effective, easy to use, a quantitative technique that can perform morphological analysis, and study certain physical and biochemical properties of the skin: not only is it able to measure skin thickness, but is also able to assess other characteristics of the skin, eg, the subcutaneous connective tissue processes, which may occur prior to changes in skin thickness.^10,11^ Using US imaging, we could detect diffuse cutaneous systemic sclerosis (dcSSc) in the very early stages of the disease, ie, less than 2 years. Compared to limited SSc and healthy controls, thicker skin and lower skin echogenicity can be seen in dcSSc, supposedly, reflecting the oedematous phase of the disease.^11,12^

As scleroderma progresses and becomes more severe over time, heel fat pad tissue becomes atrophic and more collagen accumulates in the dermis, making soft tissues stiffer and less compressible.

While this study investigated a number of biomechanical indices in patients with scleroderma as opposed to ageand BMI-matched healthy subjects, there are certain limitations that should be discussed. Firstly, heel pad properties and foot ulcers are theoretically affected by biomechanical and ischemic factors and skin thickness.

We could not calculate the effects of each of these factors separately. Another weakness was that we did not match the patients with and without digital ulcers based on their medical treatments. Therefore, the probable side-effects of drugs on the feet’s biomechanical properties were not considered. Finally, because of the cross-sectional nature of our study, we could not see the course of change in thickness and compressibility indices over time, which can be a more valuable index than a static measurement in the prediction of digital ulcers and disease progression.

## CONCLUSION

Foot ulcers are disturbing complications of scleroderma, causing great functional disability in patients and may also be associated with internal organ involvement. Given its high morbidity, our study aimed to look at the mechanical side of this catastrophic problem and to identify its predictive and risk factors before ulcers appear. Ultrasonography and conventional radiography are two inexpensive imaging modalities for evaluating soft tissue thickness and compressibility index. Given that both the latter indices change in patients with systemic sclerosis, they could be used as predictors of fat pad atrophy and elasticity in the feet. Future studies can investigate the association between microvascular changes in feet and biomechanical alterations of soft tissue. Furthermore, the effects of therapeutic agents on these quantitative changes could be possible future areas of investigation.

## ETHICAL APPROVAL

This study was in accordance with the standards of the Ethics Committee at Iran University of Medical Sciences, and in accordance with the 1964 Helsinki Declaration and its later amendments. Informed consent was obtained from all individual participants included in the study.

## Figures and Tables

**Supplementary Table.** Correlation of heel pad thickness and compressibility with demographic and baseline variable.

**Correlations**							
		Sex	Age	BMI	disease_duration_month	R_heel_unloaded_Xray	R_compressivity_index
Sex	Pearson Correlation	1	.142	.213	.147	−.142	−.056
	Sig. (2-tailed)		.209	.057	.372	.207	.619
	N	80	80	80	39	80	80
Age	Pearson Correlation	.142	1	.311^**^	.473^**^	−.001	.156
	Sig. (2-tailed)	.209		.005	.002	.993	.168
	N	80	80	80	39	80	80
BMI	Pearson Correlation	.213	.311^**^	1	.121	.279^*^	.027
	Sig. (2-tailed)	.057	.005		.465	.012	.815
	N	80	80	80	39	80	80
disease_duration_month	Pearson Correlation	.147	.473^**^	.121	1	−.434^**^	.096
	Sig. (2-tailed)	.372	.002	.465		.006	.561
	N	39	39	39	39	39	39
R_heel_unloaded_Xray	Pearson Correlation	−.142	−.001	.279^*^	−.434^**^	1	−.148
	Sig. (2-tailed)	.207	.993	.012	.006		.189
	N	80	80	80	39	80	80
R_compressivity_index	Pearson Correlation	−.056	.156	.027	.096	−.148	1
	Sig. (2-tailed)	.619	.168	.815	.561	.189	
	N	80	80	80	39	80	80

** Correlation is significant at the 0.01 level (2-tailed).

* Correlation is significant at the 0.05 level (2-tailed).

## References

[1] Eckes B Moinzadeh P Sengle G Hunzelmann N Krieg T. Molecular and cellular basis of scleroderma. J Mol Med 2014; 92:4:913–24. 25030650 10.1007/s00109-014-1190-x

[2] Nihtyanova SI Brough GM Black CM Denton CP. Clinical burden of digital vasculopathy in limited and diffuse cutaneous systemic sclerosis. Ann Rheum Dis 2008;67(1):120–3. 17660220 10.1136/ard.2007.072686

[3] Blagojevic J Bellando-Randone S Abignano G Avouac J Cometi L Czirják L Classification, categorization and essential items for digital ulcer evaluation in systemic sclerosis: a DeSScipher/European Scleroderma Trials and Research group (EUSTAR) survey. Arthritis Res Ther 2019;21(1):35. 30678703 10.1186/s13075-019-1822-1 PMC6346551

[4] Sari-Kouzel H Hutchinson CE Middleton A Webb F Moore T Griffin K Herrick AL. foot problem in patients with systemic sclerosis. Rheumatology 2001;40(4):410–3. 11312379 10.1093/rheumatology/40.4.410

[5] Poormoghim H Andalib E Jalali A Salimi-Beni M Ghafarpour GH. Foot pain and lesions in systemic sclerosis: prevalence and association with organ involvement. J Rheum Dis Treat 2019;5:076.

[6] Lamm BM Stasko PA Gesheff MG Bhave A. Normal foot and ankle radiographic angles, measurements, and reference points. J Foot Ankle Surg 2016;55(5):991e998. 27320694 10.1053/j.jfas.2016.05.005

[7] Mickle KJ Munro BJ Lord SR Menz HB Steele JR. Soft Tissue Thickness under the Metatarsal Heads is Reduced in older People with Toe Deformities. J Orthop Res 2011;29(7):1042–6. 21567451 10.1002/jor.21328

[8] Sunderkotter C Herrgott I Brückner C Moinzadeh P Pfeiffer C Comparison of patients with and without digital ulcers in systemic sclerosis: detection of possible risk factors. Br J Dermatol 2009;160(4):835–43. 19183180 10.1111/j.1365-2133.2008.09004.x

[9] Chao CY Zheng YP Cheing GL. Epidermal thickness and biomechanical properties of plantar tissues in diabetic foot. Ultrasound Med Biol 2011;37(7):1029–38. 21640473 10.1016/j.ultrasmedbio.2011.04.004

[10] Hughes M Bruni C Cuomo G Delle Sedie A Gargani L Gutierrez M The role of ultrasound in systemic sclerosis: On the cutting edge to foster clinical and research advancement. J Scleroderma Relat Disord 2021;6(2):123–32. 35386740 10.1177/2397198320970394 PMC8892934

[11] Akesson A Hesselstrand R Scheja A Wildt M. Longitudinal development of s involvement and reliability of high frequency ultrasound in systemic sclerosis. Ann Rheum Dis 2004;63(7):791–6. 15194573 10.1136/ard.2003.012146 PMC1755078

[12] Hesselstrand R Scheja A Wildt M Akesson A. High-frequency ultrasound of skin involvement in systemic sclerosis reflects oedema, extension and severity in early disease. Rheumatology (Oxford) 2008 Jan;47(1):84–7. 18077496 10.1093/rheumatology/kem307

